# Genome-wide association mapping of resistance against rice blast strains in South China and identification of a new *Pik* allele

**DOI:** 10.1186/s12284-019-0309-7

**Published:** 2019-07-15

**Authors:** Chenggang Li, Dan Wang, Shasha Peng, Yue Chen, Pin Su, Jianbin Chen, Limin Zheng, Xinqiu Tan, Jinling Liu, Yinghui Xiao, Houxiang Kang, Deyong Zhang, Guo-Liang Wang, Yong Liu

**Affiliations:** 1 State Key Laboratory of Hybrid Rice and Institute of Plant Protection, Hunan Academy of Agricultural Sciences, Changsha, 410125 China; 2 Southern Regional Collaborative Innovation Center for Grain and Oil Crops in China and College of Agronomy, Hunan Agricultural University, Changsha, 410128 Hunan China; 3 Institute of Plant Protection, Chinese Academy of Agricultural Sciences, Beijing, 100193 China; 4 Department of Plant Pathology, Ohio State University, Columbus, OH 43210 USA

**Keywords:** GWAS, Rice blast, QTL, R gene, *Pikx*

## Abstract

**Background:**

Effective management of rice blast, caused by the fungus *Magnaporthe oryzae*, requires an understanding of the genetic architecture of the resistance to the disease in rice. Rice resistance varies with *M. oryzae* strains, and many quantitative trait loci (QTLs) affecting rice blast resistance have been mapped using different strains of *M. oryzae* from different areas. However, little is known about the genetic architecture of rice resistance against the *M. oryzae* population in Hunan Province, which is a main rice production area in South China.

**Results:**

In this study, we used three isolates from Hunan Province and the rice diversity panel 1 to perform a genome-wide association study (GWAS) of blast resistance in rice. A total of 56 QTLs were identified. One of the QTLs is localized with the resistance gene *Pik* locus which confers resistance to all three isolates. Genomic sequence analysis of the resistant cultivars led to the identification of a new *Pik* allele, which we named *Pikx*. Yeast two-hybrid and co-immunoprecipitation assays between AvrPiks and Pikx confirmed that *Pikx* is a new allele at the *Pik* locus.

**Conclusions:**

Our GWAS has identified many new blast resistance QTLs. The identified new *Pik* allele *Pikx* will be useful for breeding cultivars with high resistance to blast in Hunan and other South China provinces. Further research on the relationship between *AvrPiks* and *Pikx* will provide new insights into the molecular mechanism of rice resistance to *M. oryzae*.

**Electronic supplementary material:**

The online version of this article (10.1186/s12284-019-0309-7) contains supplementary material, which is available to authorized users.

## Background

Rice is a staple food of more than half of all people worldwide (Gnanamanickam 2009). However, rice production is affected by many diseases that threaten the food security of the increasing world population. Rice blast, caused by the fungal pathogen *Magnaporthe oryzae*, is a destructive disease of rice (Valent and Chumley 1991). It typically causes an annual yield loss of 10–30% and leads to large economic losses in many countries (Skamnioti and Gurr 2009). Rice varieties with blast resistance can help control this pathogen (Hulbert et al. 2001).

Two types of resistance genes are responsible for rice blast resistance: major resistance (*R*) genes that confer race-specific resistance and quantitative trait loci (QTLs) that control partial, nonrace-specific resistance (Skamnioti and Gurr 2009). More than 100 blast resistance loci or genes have been mapped to rice chromosomes (Fang et al. 2016). Among these, only 28 *R* genes and 2 QTLs have been cloned and characterized (Xiao et al. 2017; Deng et al. 2017; Zhao et al. 2018). These cloned R genes are distributed across all 12 chromosomes except chromosome 3 (Yang et al. 2009). All of the cloned *R* genes except for *Pi-d2*, *pi21* and *Ptr* contain nucleotide-binding domain leucine-rich repeat (NLR) proteins (Liu et al. 2010; Zhao et al. 2018).

Among the cloned *R* genes, the *Pik* locus is especially important because it harbors a number of blast *R* genes used in rice breeding (Zhai et al. 2011). The *Pik* locus has at least six alleles (*Pik*, *Pikm*, *Pikp*, *Piks*, *Pikh*, and *Pi1*) that cluster on the end of the long arm of chromosome 11 (Zhai et al. 2011; Liu et al. 2014). Three of these alleles (*Pikm*, *Pikp*, and *Pik*) have been isolated and characterized (Zhai et al. 2011). Because *R* genes are highly specific to *M. oryzae* races, resistance of a single *R* gene is often rapidly overcome by the selection of compatible pathogen races (Hittalmani et al. 2000; Oliveira-Garcia and Valent 2015). In response to the rapid evolution of *M. oryzae*, the rice genome has evolved *R* gene polymorphism, which confers multiple forms of race-specific resistance (Hayashi et al. 2004). For example, the physical interaction of *Pik* alleles with specific *Avr*-*Pik* alleles can be explained by the coevolution of pathogen and host (Yoshida et al. 2009; Kanzaki et al. 2012; Wu et al. 2014). The *Pik* gene comprises two NBS-LRR genes, *Pik-1* and *Pik-2*, and the former acts as the senor for the interaction with the corresponding AvrPik protein and the latter is responsible for defense activation and signaling. A recent study showed that polymorphic residues in Pik-1 determine the resistance specificity (Carlos et al. 2018)

Genome-wide association study (GWAS) has recently been used for assessing associations between genetic markers and blast resistance in rice. GWAS was first used to identify genes underlying complex diseases in humans (Altshuler et al. 2008). With its wider use and the development of GWAS modeling (Price et al. 2006; Yu et al. 2006; Liu et al. 2016), GWAS has become a powerful approach for mapping a number of traits of rice, including agronomic traits (Huang et al. 2010; Zhao et al. 2011) and tolerance to abiotic stress (Zhu et al. 2015; Lv et al. 2016; Wang et al. 2016a, 2016b; Kaler et al. 2017). In rice, GWAS in combination with high-throughput sequencing and gene knockout techniques has been used to rapidly identify new functional genes that influence yield, heading, awn length, and other agronomic traits (Si et al. 2016; Yano et al. 2016).

In their study of resistance to 16 representative blast strains collected from all over China, researchers recently identified 30 loci associated with blast resistance using an indica rice population (Wang et al. 2014). The rice diversity panel 1 (RDP1), which comprises over 400 rice cultivars from 82 countries, was developed for GWAS of agronomical traits, and is publicly available (Zhao et al. 2011). Using RDP1, our team previously performed an association study of rice resistance to blast isolates from Asia (China, India, the Philippines, and South Korea) and the Americas (Colombia), which led to the identification of 97 loci associated with blast resistance along with two new *Pi5* alleles (Kang et al. 2016). Using eight *M. oryzae* isolates from four African counties, 31 rice genomic regions associated with blast resistance were identified on another study (Mgonja et al. 2016). Similarly, our team analyzed QTLs associated with blast resistance in the field and identified 16 loci associated with field blast resistance using RDP1 (Zhu et al. 2016).

In the current study, we performed GWAS on rice blast using the 234 RDP1 cultivars and three isolates of the pathogen from the major rice production regions in Hunan Province, China. A total of 56 QTLs associated with blast resistance were identified in the rice genome. Only one QTL associated with resistance to all three isolates was found, and it was localized with the known *R* gene *Pik* locus. Genotype analysis of the significantly associated SNP-11.27701887 on the *Pik* locus in 234 cultivars confirmed the association between genotype and resistance to the three isolates. In addition, sequence analysis and protein-protein interaction analysis indicated that this gene is a previously unreported allele on the *Pik* locus. The new allele was named *Pikx*.

## Methods

### Plant and fungal materials

**Fig. 1:**
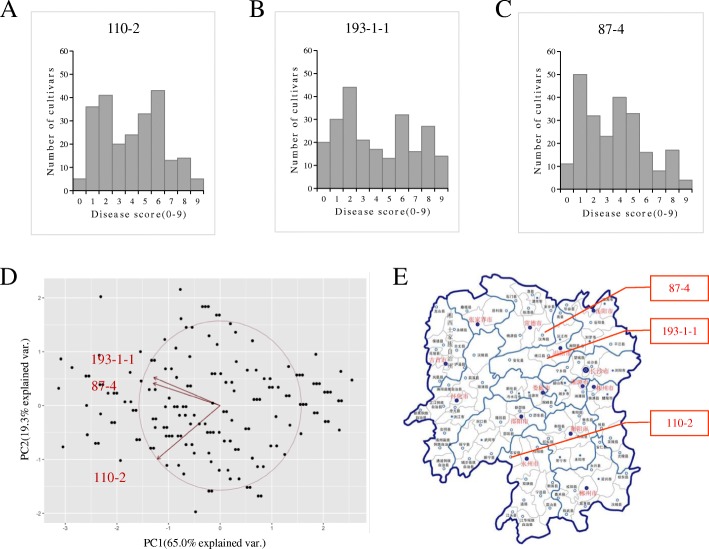
The information of three *M. oryzae* isolates and blast disease scores of the 234 RDP1 cultivars. **a**-**c** The distribution of disease scores of the 234 RDP1 cultivars against isolates 110-2, 193-1-1 and 87-4. **d** PCA of resistance phenotypes of the 234 RDP1 cultivars to the three isolates. **e** Geographic distribution of three *M. oryzae* isolates in Hunan Province Map

A total of 234 rice accessions from RDP1 were used, and these comprised 59 *tropic japonica* (TRJ), 35 *temperate japonica* (TEJ), 53 *indica* (IND), 45 *aus* (AUS), 10 *aromatic* (ARO), and 32 admixture (ADMIX) accessions (Zhao et al. 2011). Fifteen seeds of each accession were germinated and sowed in small pots with soil. The rice seedlings were grown in a growth chamber under controlled conditions (26 °C, 75% relative humidity, and a 10 h light/14 h dark photoperiod). Three *M. oryzae* isolates, 110–2, 193–1-1, and 87–4, were collected in Dong’an County, Taojiang County, and Hanshou County, respectively, in Hunan Province (Fig. 1e). The *M. oryzae* isolates were cultured on an oat medium to obtain conidia for inoculation of 3-week-old rice seedlings.

### Blast resistance phenotyping of rice cultivars

*M. oryzae* conidia at a spore concentration of 1 × 10^5^ were sprayed onto rice leaves as described by Park et al. (2012). Each cultivar was inoculated with the three isolates in three replications. Approximately 7 days after inoculation with *M. oryzae*, disease was scored on a scale from 0 to 9 based on the size and area ratio of lesions as described previously (Kang et al. 2016). The disease score for each pot was measured, and the average values of the three replications were used to generate a data matrix.

### GWAS of rice blast resistance

The 277,524 SNP genotypes of the 234 RDP1 accessions were generated from the 700 K SNP RDP1 genotypes using P-link with the criterion of minor allele frequency ≥ 5%. GWAS was performed with the software EMMAX (Kang et al. 2010, http://csg.sph.umich.edu/kang/emmax/) using the 277,524 SNP genotypes and the phenotype dataset. SNPs with -Log_10_(*P-*value) ≥ 4.0 were considered to have significant associations. Manhattan and Q-Q plots were generated with the R environment (https://cran.r-project.org/web/packages/qqman).

### Identification of QTLs and selection of candidate genes

QTLs were identified by using the Nipponbare genome as a reference, and candidate genes from the 200 kb interval regions around the peak SNPs were selected. All of the reported R or defense-related genes in plants, including NLR, serine-threonine kinase, and transcription factor, were considered for selection of candidate genes (Liu et al. 2014; Li et al. 2017).

### Characterization and validation of the functions of candidate genes

The association between QTL50 and blast resistance was confirmed based on the analysis of the genotype of the significantly associated SNPs in the 234 cultivars. The *Pik* alleles in rice and *AvrPik* alleles in *M. oryzae* were amplified using the *Pik* or *AvrPik* specific primers, respectively, (Additional file 1: Table S1) and the PCR products were sequenced.

The yeast-two hybrid (Y2H) system and co-immunoprecipitation (Co-IP) assay were used to confirm the interaction between the R and Avr proteins. The protocols followed those of the Clontech Handbook for Y2H and the Co-IP method previously described Wang et al. (2016a, 2016b). The signal peptide-truncated cDNA fragments of *AvrPik-A, −C, −D*, and *-E* were synthesized by Genereate Ltd. and were then inserted into the pGBKT7 (for Y2H) vector and into the pYBA1152 (for Co-IP) vector. Fragments of *Pikx-1-cc* that were amplified from the cDNA of rice seedlings of cultivar NSFTV_131 using PrimeSTAR GXL DNA polymerase (Takara) were cloned into pGADT7 (for Y2H) and PYBA1144 (for Co-IP) using specific primers (Additional file 1: Table S1).

## Results

### Resistance of the 234 RDP1 cultivars to the three *M. oryzae* isolates

The distribution of blast disease scores of the 234 cultivars inoculated with the three *M. oryzae* isolates is shown in Fig. 1a, b, and c. Among the inoculated rice cultivars, 11 were highly resistant (disease scores ≤1), and 14 were highly susceptible (disease scores ≥8) to all the three isolates (highlighted with yellow and blue, respectively, in Additional file 2: Table S2). Principal component analysis (PCA) showed that the levels of resistance of the cultivars to the three isolates were diverse (Fig. 1d). However, the disease scores of the cultivars to isolates 193–1-1 and 87–4 were clustered perhaps because the two isolates were collected in fields that were near each other and that might therefore contain similar avirulence genes.

### Association mapping of rice QTLs linked to resistance to *M. oryzae*

**Fig. 2:**
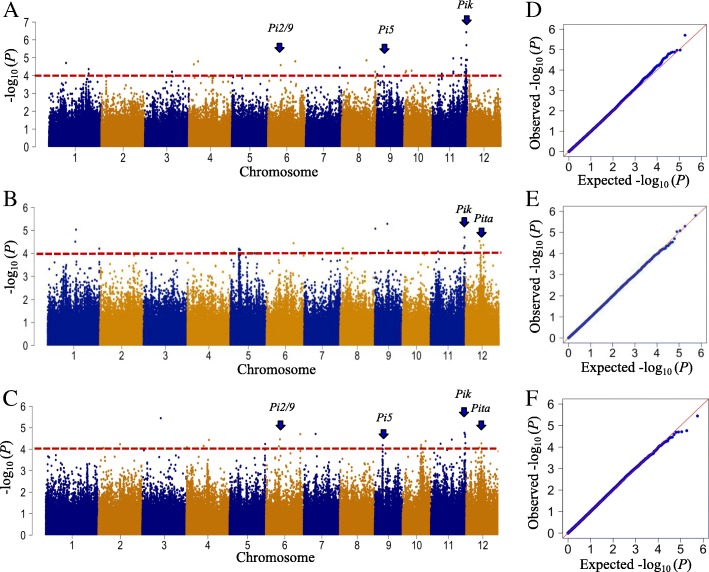
Genome-wide association studies of rice resistance to three *M. oryzae* isolates. **a**. Manhattan plot of GWAS results for rice resistance to 110-2. **b**. Manhattan plot of GWAS results for rice resistance to 193-1-1. **c**. Manhattan plot of GWAS results for rice resistance to 87-4. The X-axis indicates the SNPs physically mapped on 12 chromosomes. **d, e** and **f**. Quantilequantile plots of expected and observed -log10(p-value) to three isolates, respectively

To identify genomic regions that are associated with blast resistance to the three isolates, we performed a GWAS using the disease scores and the 700 K SNP genotypes of the inoculated cultivars. A total of 56 QTLs associated with blast resistance to the three isolates were detected in the rice genome (−Log_10_ *P* ≥ 4.0) (Fig. 2). Among the QTLs, 24 loci were associated with resistance to isolate 110–2, 32 were associated with resistance to isolate 87–4, and 22 were associated with resistance to isolate 193–1-1 (Additional file 3: Table S3). Some of the loci were associated with resistance to two isolates, i.e.*,* three loci were associated with resistance to both 110–2 and 87–4, two loci to both 110–2 and 193–1-1, and one locus to 87–4 and 193–1-1. Among all of the mapped QTLs, 16 were located at sites of five previously cloned resistance genes, and 40 were loci identified for the first time in this study. Interestingly, seven QTLs (46–52) were located in the *Pik* region on chromosome 11 (Fjellstrom et al*.* 2004) (Additional file 3: Table S3). Among them, QTL48, QTL50, and QTL51 were associated with resistance to all three isolates. In addition, seven of the QTLs (QTL21, QTL48, QTL49, QTL50, QTL51, QTL52 and QTL53) were also identified in a previous study (Kang et al. 2016) (Additional file 4: Table S4). In particular, the *Pik* locus was identified in both Kang et al. (2016) and the present study.

### QTL50 is linked to the *Pik* locus

**Fig. 3:**
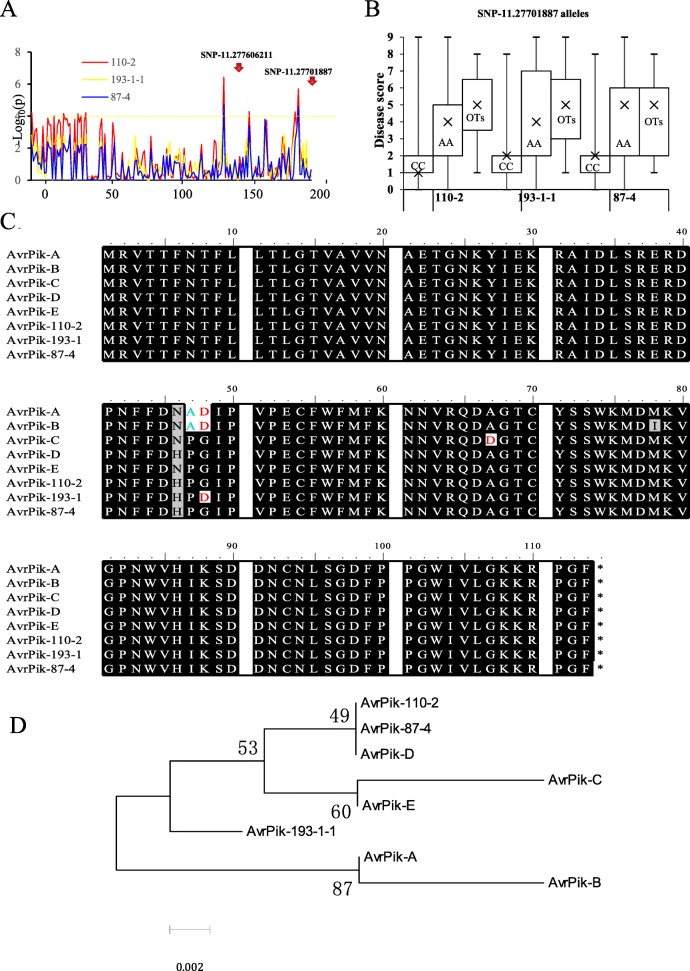
Relationship between QTL50 and *Pik* and the *AvrPik* sequences in the three isolates **a** SNP-11.27701887 is the most significant SNP that is associated with resistance to all three *M. oryze* isolates in QTL50. **b** SNP-11.27701887 alleles distribution in the 234 RDP1 cultivars. CC, AA and OTs represent the genotype AA, CC and other type of SNP-11.27701887, respectively. **c** Sequence alignment of the AvrPik amino acid sequences using ClustalW. Shading fonts indicate common amino acid residues in the alleles. **d** Phylogeny tree of the *AvrPik* genes from three *M. oryze* isolates

To determine the relationship between QTL48/QTL50/QTL51 and *Pik*, we identified 14 significant SNPs that were linked to the three QTLs (Additional file 3: Table S3) in the 200-kb region. Among them, SNP-11.27606211 and SNP-11.27701887 at QTL50 were tightly associated with resistance to the three isolates (Fig. 3a). Haplotype analysis of the 234 RDP1 cultivars indicated that SNP-11.27701887 had a strong association with the resistance to the three isolates (Fig. 3b). In addition, we sequenced the *AvrPik* genes from the three isolates using *AvrPik*-specific primers (Additional file 1: Table S1). Sequence analysis showed that 110–2 and 87–4 harbor *AvrPik-D*, 193–1-1 harbors *Avr* gene similar to *AvrPik-D* and *AvrPik-E* (Fig. 3c, d), suggesting that QTL50 may be an allele at the *Pik* gene that can recognize the three *AvrPik* genes in the fungus.

### Identification and validation of the new allele at the *Pik* locus

**Fig. 4:**
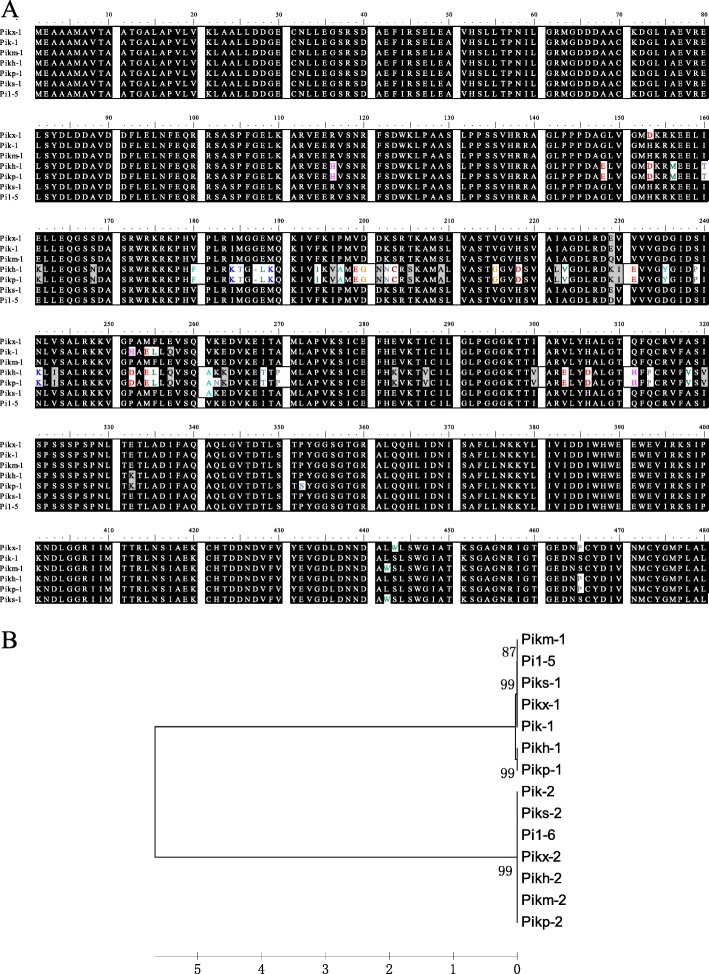
Sequence comparison between *Pikx* and other *Pik* alleles **a** Sequence alignment of the Pik cc domain amino acid sequences using ClustalW. Shading fonts indicate common amino acid residues in the alleles. **b** Phylogenetic analysis of Pik allele protein sequences using MEGA 5. The tree was generated using UPGMA. Numbers stand for bootstrap values from 1000 replicates

To determine whether the gene at QTL50 is a new allele of *Pik*, we sequenced 11 blast-resistant rice cultivars (NSFTV_5, 45, 53, 93, 119, 131, 160, 321, 344, 349, and 622) by PCR amplification (Additional file 2: Table S2, highlighted with yellow). Five of them are ARO, 2 are IND, 2 are AUS and 2 are ADMIX. Among the 11 cultivars, 5 contain the AA type and 5 contain the CC type at SNP-11.27701887 (AA type is the blast resistant type as shown in Fig. 3b). Two of these cultivars, NSFTV_622 (ADMIX, AA type at SNP-11.27701887) and NSFTV_131(AUS, AA type at SNP-11.27701887), were found to harbor the *Pik* alleles. Sequence analysis of the predicted amino acids of the two genes indicated that an allele from cultivar NSFTV_622 was the same as the known *R* gene *Pik-s*, and that another allele from cultivar NSFTV_131 was a new allele, which we named *Pikx* and which has a unique amino acid at 443S in Pikx-1 (substitution with W) (Fig. 4a). Phylogenetic analysis showed that all of the Pik allele-1 proteins are highly polymorphic but the Pik allele-2 proteins are less polymorphic (Fig. 4b). Pikx-1 was clustered in the same clade with Pikm-1, Pi1–5, Piks-1 and Pik-1 (Fig. 4b).

**Fig. 5:**
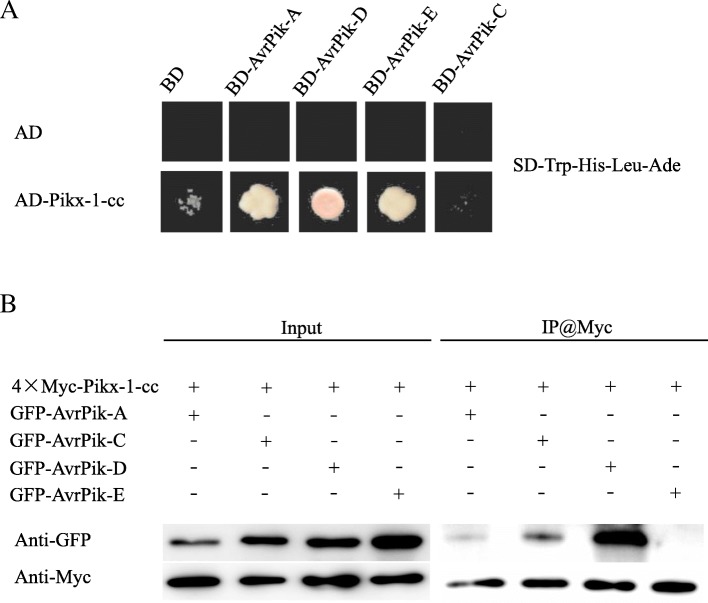
Interaction detection between Pikx and four AvrPik proteins in yeast and *in planta* **a** Y2H analysis of the interaction between Pikx-1 cc domain and four AvrPik alleles. **b** Co-IP analysis of the interaction between Pikx-1 cc domain and four AvrPik alleles in *N. benthamiana*

A previous study reported that Pik proteins physically interact with AvrPik by the Pik-1 cc domain, and that the binding specificity between Pik-1 and AvrPik determines the recognition specificity between Pik and Avr-Pik (Kanzaki et al. 2012). To determine the relationship between Pikx and AvrPiks, we performed Y2H and Co-IP assays in order to detect the interaction between Pikx and four AvrPik alleles (A, C, D, and E). The Y2H assay showed that AvrPik-A, −D, and -E strongly interacted with Pikx-1-cc (Fig. 5a). In the Co-IP assay, however, Pikx-1-cc strongly interacted only with AvrPik-E and weakly interacted with AvrPik-A and -D (Fig. 5b). Pikm was reported to interact with AvrPik-A, −C, and -D (Kanzaki et al. 2012). Therefore, Pikx is similar to Pikm in terms of its interaction with the AvrPik alleles.

## Discussion

Given the genomic instability of *M. oryzae* populations, many resistance genes in rice are short-lived, and the identification of new blast resistance genes is therefore essential for rice breeding programs. The identification of new resistance resources can be greatly facilitated by GWAS. Phenotypic evaluations in GWAS can usually be performed within 1 year with multiple replications in the greenhouse or field, whereas conventional bi-parental QTL mapping usually requires 2 or more years for population development and phenotype evaluation. In addition, the markers used in GWAS are high throughput and high density, and the associated markers identified in the analysis can be closer to the target genes than do those identified in conventional QTL mapping. As a consequence, GWAS provides a rapid way to identify functional genes in rice. To study blast resistance in rice, Kang et al. (2016) performed GWAS of the RDP1 population and identified 97 loci associated with blast resistance, 82 of which had not been previously identified. Using GWAS in the current study, we identified 56 QTLs involved in blast resistance. Among them, 40 have not been previously identified. These results demonstrate that majority of the QTLs identified in the current study are specific to the three *M. oryzae* strains from Hunan.

Many QTLs for rice blast resistance have been previously reported (Liu et al. 2010; Liu et al. 2014). Among the loci previously identified in different mapping populations, several were also identified in our study (Deng et al. 2006; Hayashi et al. 2004; Hittalmani et al. 2000; Kang et al. 2016). For example, five loci were detected in the same rice population when inoculated with different blast isolates (Additional file 4: Table S4) (Kang et al. 2016). One of the five common loci is *Pik* on chromosome 11, which has been frequently studied in the last several years. Some of the alleles at the *Pik* locus such as *Pik*, *Pik-1*, *Pik-p*, and *Pik-m* have been isolated and characterized (Ashikawa et al. 2008; Yuan et al. 2011; Zhai et al. 2011; Hua et al. 2012). In this study, we identified a new allele, *Pikx*, that is significantly associated with rice blast resistance at the *Pik* locus. Because it confers resistance to the three isolates collected in Hunan, we speculate that this new gene may be useful for rice breeding against the *M. oryzae* population in Hunan and other provinces in South China. The function of this gene in blast resistance will be confirmed by rice transformation in the future.

Our Y2H and CoIP assays also indicated that Pikx-1 cc, which is similar to Pik-m-1-cc reported in Kanzaki et al. (2012), can interact with AvrPik-A, −D, and -E. These interactions may be possible because the key sites of the Avr-R-recognition domain HMA in the Pikx-1 cc are identical to those of the Pik-m-1 cc domain (Maqbool et al. 2015). Avr-Pik is a 113 amino acid protein with a 21 amino acid signal peptide at its N terminus (Yoshida et al. 2009). The latter study identified five alleles of *Avr-Pik* (*Avr-Pik*-*A*, *B*, *C*, *D*, and *E*) in 21 isolates of *M. oryzae* from Japan and found that the five *Avr-Pik* alleles differ from one other by a total of five DNA substitutions, all of which cause amino acid changes. According to Yoshida et al. (2009), the *Avr-Pik-D* allele is likely the ancestral allele from which the *Avr-Pik-E*, −*C*, −*A*, and -*B* alleles are derived. As noted earlier, Yoshida et al. (2009) found *Avr-Pik-B* in an isolate (isolate 9505–3) from Japan. In the present study, the Y2H assay showed that AvrPik-A, −D, and -E strongly interact with Pikx-1-cc (Fig. 5a). In the Co-IP assay, however, Pikx-1-cc strongly interacted only with AvrPik-E and weakly interacted with AvrPik-A and -D (Fig. 5b). The reason for the difference warrants further investigation.

## Additional files

Additional file 1: **Table S1.** Primers used in this study. (XLSX 11 kb)

Additional file 2: **Table S2.** Phenotype of 234 RDP1 varieties against Magnaporthe oryzae isolates. (XLS 61 kb)

Additional file 3: **Table S3.** The regions associated with the blast resistance QTLs to three *M. oryzae* isolates. (XLSX 19 kb)

Additional file 4: **Table S4.** The shared loci identified in the present study and previous study. (XLSX 13 kb)

## Data Availability

The datasets supporting the conclusions of this article are provided within the article and its additional files.
